# Mortality risk of black women and white women with invasive breast cancer by hormone receptors, HER2, and p53 status

**DOI:** 10.1186/1471-2407-13-225

**Published:** 2013-05-04

**Authors:** Huiyan Ma, Yani Lu, Kathleen E Malone, Polly A Marchbanks, Dennis M Deapen, Robert Spirtas, Ronald T Burkman, Brian L Strom, Jill A McDonald, Suzanne G Folger, Michael S Simon, Jane Sullivan-Halley, Michael F Press, Leslie Bernstein

**Affiliations:** 1 Division of Cancer Etiology, Department of Population Sciences, Beckman Research Institute, City of Hope, Duarte, CA, 91010, USA; 2 Division of Public Health Sciences, Fred Hutchinson Cancer Research Center, Seattle, WA, 98109, USA; 3 Division of Reproductive Health, Centers for Disease Control and Prevention, Atlanta, GA, 30333, USA; 4 Department of Preventive Medicine, University of Southern California, Los Angeles, CA, 90033, USA; 5 Pathology, Keck School of Medicine, University of Southern California, Los Angeles, CA, 90033, USA; 6 Formerly Contraceptive and Reproductive Health Branch, Center for Population Research, National Institute of Child Health and Development, Bethesda, MD, 20892, USA; 7 Department of Obstetrics and Gynecology, Baystate Medical Center, Springfield, MA, 01199, USA; 8 Center for Clinical Epidemiology and Biostatistics, Department of Biostatistics and Epidemiology, University of Pennsylvania School of Medicine, Philadelphia, PA, 19104, USA; 9 Karmanos Cancer Institute, Department of Oncology, Wayne State University, Detroit, MI, 48201, USA

**Keywords:** Breast cancer, Mortality, Racial disparity, Triple negative, Luminal A, ER, PR, HER2, p53

## Abstract

**Background:**

Black women are more likely than white women to have an aggressive subtype of breast cancer that is associated with higher mortality and this may contribute to the observed black-white difference in mortality. However, few studies have investigated the black-white disparity in mortality risk stratified by breast cancer subtype, defined by estrogen receptor (ER), progesterone receptor (PR) and human epidermal growth factor receptor 2 (HER2) status. Furthermore, it is not known whether additional consideration of p53 protein status influences black-white differences in mortality risk observed when considering subtypes defined by ER, PR and HER2 status.

**Methods:**

Four biomarkers were assessed by immunohistochemistry in paraffin-embedded breast tumor tissue from 1,204 (523 black, 681 white) women with invasive breast cancer, aged 35–64 years at diagnosis, who accrued a median of 10 years’ follow-up. Multivariable Cox proportional hazards regression models were fit to assess subtype-specific black-white differences in mortality risk.

**Results:**

No black-white differences in mortality risk were observed for women with triple negative (ER-negative [ER-], PR-, and HER2-) subtype. However, older (50–64 years) black women had greater overall mortality risk than older white women if they had been diagnosed with luminal A (ER-positive [ER+] or PR+ plus HER2-) breast cancer (all-cause hazard ratio, HR, 1.88; 95% confidence interval, CI, 1.18 to 2.99; breast cancer-specific HR, 1.51; 95% CI, 0.83 to 2.74). This black-white difference among older women was further confined to those with luminal A/p53- tumors (all-cause HR, 2.22; 95% CI, 1.30 to 3.79; breast cancer-specific HR, 1.89; 95% CI, 0.93 to 3.86). Tests for homogeneity of race-specific HRs comparing luminal A to triple negative subtype and luminal A/p53- to luminal A/p53+ subtype did not achieve statistical significance, although statistical power was limited.

**Conclusions:**

Our findings suggest that the subtype-specific black-white difference in mortality risk occurs mainly among older women diagnosed with luminal A/p53- breast cancer, which is most likely treatable. These results further suggest that factors other than subtype may be relatively more important in explaining the increased mortality risk seen in older black women.

## Background

Although mortality following breast cancer diagnosis has decreased substantially in the United States over the last three decades**,** a large black-white difference remains. Black women have higher risk of death after breast cancer diagnosis than white women [1,2] and are more likely than white women to have an aggressive subtype of breast cancer that is associated with a higher mortality [3], which could contribute to the observed black-white mortality difference. However, only a few studies have investigated the black-white disparity in mortality risk by breast cancer subtype as defined by estrogen receptor (ER), progesterone receptor (PR), and human epidermal growth factor receptor 2 (HER2) status [4-7]. Furthermore, little is known whether additional consideration of p53 protein status has any influence on black-white differences in mortality risk within subtype strata.

Breast cancer is a heterogeneous disease; its subtypes have been classified as triple negative (TN) (ER-negative [ER-], PR-, and HER2-), luminal A (ER-positive [ER+] or PR+ plus HER2-), luminal B (ER+ or PR+ plus HER2+), and HER2-enriched (ER-/PR-/HER2+) subtype [8-16]. Gene expression studies using cDNA microarray technology show that TN breast cancers are often characterized by a “basal-like” molecular profile [17], characteristic of the basal epithelial cell layer, including high level expression of HER1 and/or genes encoding cytokeratins 5/6 [3]. Because cDNA microarray technology is not yet available clinically for identifying basal-like subtype, the TN subtype has become a commonly used proxy for the “basal-like” subtype in clinical and epidemiologic studies, despite the fact that TN subtype and basal-like subtype are discordant in 20-30% of cases [17,18].

TN breast tumors, which account for 10-25% of all invasive breast cancers [19,20], have poorer prognosis than luminal A, the most common subtype [8,9,13]. While ER+ breast cancers respond favorably to anti-estrogen therapy and HER2+ breast cancers respond favorably to trastuzumab therapy [20,21], no targeted therapies currently exist for TN breast cancer. Studies have consistently shown that TN breast cancers comprise a higher proportion of breast cancers in black women than white women [3,4,11,22-24]. However, little research has been done examining the extent to which black-white mortality differences exist within each specific breast cancer subtype. Two studies reported that the black-white differences in all-cause mortality [4] and breast cancer-specific mortality [6] were limited to the TN subtype. A third study reported that the crude all-cause mortality risk was greater among black women than white women irrespective of the subtypes defined by ER, PR, and HER2 status [7]. The Carolina Breast Cancer Study found instead, that the black-white differences in breast cancer-specific mortality occurred among women diagnosed with luminal A breast cancer, but not among those diagnosed with basal-like breast cancer [5].

*p*53 is a tumor suppressor gene, which encodes the p53 protein [25,26]. p53 protein is involved in gene transcription, DNA synthesis/repair, genomic plasticity and programmed cell death [27]. Mutations in *p*53 have been identified in approximately 15-35% of breast cancers [28-30] and are associated with resistance to chemotherapy, radiotherapy [31] and poor prognosis [32]. *p*53 mutations occur more frequently in breast cancers of black women than in those of white women [33] and these mutations are more common in breast cancers that are ER-/PR- [34], TN [35], or basal-like [3,34] than in breast cancers that are ER+ or PR+. *p*53 mutations, especially missense mutations, are highly correlated with the p53 protein overexpression in tumor tissue [36,37]. One epidemiologic study examined the effect of p53 status on all-cause morality for African American (AA) women and non-AA women, respectively, and found that having a p53+ tumor adversely affected prognosis among AA women but not non-AA women after controlling for multiple variables including the individual status of ER, PR and HER2 or subtype as determined by 3 or 5 marker panels. No analyses were reported on whether the overexpression status of p53 protein impacted the black-white disparity in mortality within strata of breast cancer subtype [7].

We have previously shown that white women with invasive breast cancer participating in the Women’s Contraceptive and Reproductive Experiences (CARE) Study who had higher body mass index (BMI) had higher mortality risk than those with a normal (not overweight) BMI; but this association did not hold for black women [38]. Here, we determine the extent to which black-white differences in breast cancer-specific and all-cause mortality differ for TN, luminal A, luminal B, and HER2-enriched breast cancers in a substudy conducted at two participating study sites where tumor tissue was collected. We then assess whether any black-white mortality differences that existed for the two common breast cancer subtypes, TN and luminal A, are affected by p53 protein expression status.

## Methods

### Study population and data collection

The participants for this analysis are women from two study sites, Detroit and Los Angeles (LA), participating in the Women’s CARE Study, a population-based case–control study designed to examine risk factors for invasive breast cancer among US-born black women and white women including those of Hispanic ethnicity [39]. The Women’s CARE Study selected a stratified (by age group) random sample of women aged 35 to 64 years who were newly diagnosed with histologically confirmed incident invasive breast cancer (International Classification of Diseases for Oncology codes C50.0–C50.9) between July 1994 and April 1998. Black women were oversampled to maximize their numbers in the study, and white women were sampled to provide approximately equal numbers of women in each 5-year age category (from 35 to 64 years). Race was based on participants’ self-identification. From the two study sites, the Women’s CARE Study recruited 1,921 breast cancer patients (Detroit: 679, LA: 1,242). These two study sites were selected to collect tumor tissue samples based on representative case participants in the Women’s CARE Study and the ability to obtain tumor tissue samples. All participants provided written informed consent. The study protocol was approved by the Institutional Review Boards at the University of Southern California (IRB#: HS-923048), the Karmanos Comprehensive Cancer Center at Wayne State University (IRB#: WSU HIC# H 04-09-96(M05)-FB), the Centers for Disease Control and Prevention (IRB#: 1862), and the City of Hope (IRB#: 08098).

### Assessment of biomarkers

Paraffin-embedded tumor blocks were obtained from pathology laboratories where diagnoses were made for 1,333 participating breast cancer cases (Detroit: 414, LA: 919), approximately 80% of those requested. Tumor blocks were carefully reviewed and evaluated in the centralized pathology laboratory of Dr. Michael F. Press at the University of Southern California.

We excluded 127 case samples because the tumor blocks contained only carcinoma *in situ* (n = 56) or no tumor tissue (n = 46); had insufficient tissue for assay (n = 3); had other problems (n = 14); or only hematoxlin-and-eosin stained tissue sections were received (n = 8). The expression of ER, PR, HER2, and p53 was determined for the remaining 1,206 samples (Detroit: 367, LA: 839).

The expression of ER and PR was determined using previously published immunohistochemistry (IHC) methods [40,41]. Immunostaining results for ER and PR expression were interpreted in a blind fashion and scored semiquantitatively on the basis of the visually estimated percentage of positively stained tumor cell nuclei. At least 100 tumor cells were examined for each specimen; ≥ 1% immunostained tumor cell nuclei was considered positive for ER and PR status [42].

HER2 expression was determined by IHC using the 10H8 monoclonal antibody [43,44] to assess HER2 membrane protein immunostaining. No (0) or weak (1+) membrane immunostaining was considered low HER2 expression (HER2-). Moderate (2+) or strong membrane immunostaining (3+) was considered HER2 overexpression (HER2+) based on previous validation results from the same pathology laboratory, indicating over 90% specimen samples scored as 2+ (80.6%) or 3+ (98.9%) by 10H8-IHC showed HER-2 gene amplification by fluorescent in situ hybridization (FISH) analysis [43].

The expression of p53 protein was determined by IHC using the monoclonal mouse antibodies DO7 (Oncogene Science, Inc. Cambridge, MA) and BP 53-12-1 (Biogenex) to measure p53 nuclear protein immunostaining. Based on findings from previous studies, comparing p53 mutations in exons 2–11 with p53 protein expression levels [37,45], ≥10% nuclear staining for p53 protein was deemed positive [46].

### Tumor characteristics from SEER

The Women’s CARE Study collected tumor stage, tumor histologic grade, and other tumor characteristics. We excluded two more women because they were missing information on tumor stage, resulting in the final sample size of 1,204 (523 black, 681 white) women for the analyses.

### Vital status follow-up

Women were followed up annually for vital status, date of death and cause of death using standard SEER follow-up procedures. Women from Detroit were followed through December 31, 2004; follow-up extended until December 31, 2007 in LA.

### Statistical analyses

We used Pearson Chi-squared tests to compare frequency distributions of categorical variables between black women and white women.

Adjusted estimates of the hazard ratio (HR) of death, a measure of relative risk, and its 95% confidence interval (CI), comparing black women to white women, were calculated for each breast cancer subtype of interest using Cox proportional hazards regression models [47]. Two Cox proportional hazards regression models were applied. In Model 1, we used age (in days) at diagnosis and at death or end of follow-up as the time scale, and stratified by single years of age at diagnosis and adjusted for study site. In the analyses of breast cancer-specific mortality (International Classification of Diseases codes ICD9-174, ICD10-C50), women who died from other causes were censored on their dates of death. In Model 2, we additionally adjusted for tumor stage. Tumor grade was not included in Model 2 since it did not cause more than a 10% change in any of the risk estimates. We conducted the analyses for all women and separately for two age groups (younger: 35–49, older: 50–64 years at diagnosis). Homogeneity of race-specific HRs across different subtypes was evaluated using a Z test of the differences in adjusted log race-specific HRs divided by the square root of the sum of the variances of the two race-specific log HRs [48]. Since 9 black women and 73 white women reported Hispanic ethnicity, we repeated all the analyses after excluding these 82 women. Our results remained similar. Therefore, we present the results based on the analyses of all participants.

Kaplan-Meier breast cancer-specific curves [49] were constructed to demonstrate black-white survival differences observed in older women with luminal A invasive breast cancer.

We considered a two-sided *P* value less than 0.05 as statistically significant when testing for homogeneity of HRs across subtypes of breast cancer. All statistical analyses were performed using SAS version 9.2 software (SAS Institute, Cary, NC).

## Results

### Study population characteristics

During a median follow-up of 10 years (9.9 years and 10.0 years for black women and white women, respectively), 272 (141 black and 131 white) women died specifically from breast cancer and 63 (39 black and 24 white) women died from other causes. Compared with white women, black women were more likely to be diagnosed with ER-, PR-, TN, p53+, non-localized, or higher grade tumors (all *P* < 0.001, Table 1). The frequency distribution of HER2 in black women was not statistically significantly different from that of white women overall (*P* = 0.16) or in younger women 35 to 49 years of age (*P* = 0.98), whereas older black women 50 to 64 years of age were more likely to be diagnosed with HER2+ tumors than white women in the same age group (*P* = 0.04).

**Table 1:** Percent distribution of selected characteristics at diagnosis in 1,204 women with invasive breast cancer

	**All women**			**Younger women**			**Older women**		
				**(ages 35–49 y)**			**(ages 50–64 y)**		
	**White**	**Black**	***P***^**a**^	**White**	**Black**	***P***^**a**^	**White**	**Black**	***P***^**a**^
	**n=681**	**n=523**		**n=345**	**n=272**		**n=336**	**n=251**	
Study site			0.01			0.07			0.09
Los Angeles	66.7	73.2		64.9	71.7		68.5	74.9	
Detroit	33.3	26.8		35.1	28.3		31.6	25.1	
Age at diagnosis, years			0.004			<0.001			
35-39	21.4	14.2		42.3	27.2		-	-	
40-44	15.9	19.5		31.3	37.5		-	-	
45-49	13.4	18.4		26.4	35.3		-	-	
50-54	18.7	17.6		-	-		37.8	36.7	0.42
55-59	14.5	16.4		-	-		29.5	34.3	
60-64	16.2	14.0		-	-		32.7	29.1	
ER status			<0.001			0.05			<0.001
ER-	36.9	48.8		46.4	54.4		27.1	42.6	
ER+	63.1	51.2		53.6	45.6		72.9	57.4	
PR status			<0.001			<0.001			<0.001
PR-	38.5	52.4		42.6	54.0		34.2	50.6	
PR+	61.5	47.6		57.4	46.0		65.8	49.4	
HER2 status			0.16			0.98			0.04
HER2-	83.3	80.1		81.2	81.3		85.4	78.9	
HER2+	16.7	19.9		18.8	18.7		14.6	21.1	
Subtypes defined by ER/PR/HER2			<0.001			0.12			<0.001
TN	23.8	33.5		30.4	39.0		17.0	27.5	
Luminal A	59.5	46.7		50.7	42.3		68.5	51.4	
Luminal B	9.8	10.3		11.0	9.9		8.6	10.8	
HER2-enriched	6.9	9.6		7.8	8.8		6.0	10.4	
p53 status			<0.001						0.003
p53-	77.0	65.8		72.5	61.0	0.003	81.6	70.9	
p53+	23.0	34.2		27.5	39.0		18.4	29.1	
Subtypes defined by ER/PR/HER2/p53			<0.001			0.03			<0.001
TN/p53-	14.4	16.6		18.0	18.8		10.7	14.3	
TN/p53+	9.4	16.8		12.5	20.2		6.3	13.2	
Luminal A/p53-	49.9	37.7		41.5	31.3		58.6	44.6	
Luminal A/p53+	9.5	9.0		9.3	11.0		9.8	6.8	
Luminal B/p53-	8.1	6.0		8.4	5.2		7.7	6.8	
Luminal B/p53+	1.8	4.4		2.6	4.8		0.9	4.0	
HER2-enriched/p53-	4.6	5.5		4.6	5.9		4.5	5.2	
HER2-enriched/p53+	2.4	4.0		3.2	2.9		1.5	5.2	
Stage			<0.001			0.007			0.001
Localized	63.1	51.1		56.5	45.6		69.9	57.0	
Non-localized	36.9	49.0		43.5	54.4		30.1	43.0	
Grade			<0.001			0.006			<0.001
Low	12.5	8.6		9.6	6.6		15.5	10.8	
Intermediate	63.1	53.0		58.6	49.3		67.9	57.0	
High	24.4	38.4		31.9	44.1		16.7	32.3	

^a^*P* ascertained from Pearson χ^2^ test. Abbreviations: ER, estrogen receptor; PR, progesterone receptor; HER, human epidermal growth factor receptor; TN, triple negative. Note: TN = ER-/PR-/HER2-, Luminal A = ER+ or PR+ plus HER2-, Luminal B = ER+ or PR+ plus HER2+, HER2-enriched = ER-/PR-/HER2+.

### Black-white difference in breast cancer-specific mortality

**Figure 1:**
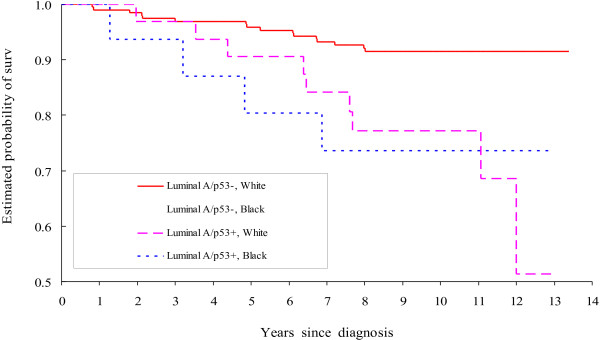
Kaplan-Meier breast cancer-specific survival of older black women vs. older white women diagnosed with luminal A invasive breast cancer sub-typed by p53.

After controlling for age at diagnosis and study site, black-white differences for breast cancer-specific mortality risk were observed among women diagnosed with luminal A breast cancer (HR, 1.52; 95% CI, 1.01 to 2.28), but not among those diagnosed with TN breast cancer (HR, 1.21; 95% CI, 0.81 to 1.83, Table 2). The magnitude of race-specific HR estimates for other subtypes (luminal B and HER2-enriched) was at least as great as that for luminal A but due to small numbers for these subtypes (and thus few deaths), 95% CIs included 1.0. Analyses by age group at diagnosis (35–49 versus 50–64 years) showed that the black-white differences in breast cancer-specific mortality predominately existed among older women with luminal A tumors (HR, 2.07; 95% CI, 1.16 to 3.70), but not in younger women diagnosed with luminal A tumor or among women diagnosed with TN tumor regardless of age group. When older women were further stratified by p53 protein expression, the black-white difference in mortality risk was observed among those with luminal A tumors that were p53- (HR, 2.53; 95% CI, 1.27 to 5.04, Figure 1).

**Table 2:** Adjusted HRs of breast cancer-specific mortality associated with race (black women vs. white women)

	**White**		**Black**		**Model 1**^**a**^		**Model 2**^**a,b**^	
	**Person-years**	**Death (No.)**	**Person-years**	**Death (No.)**	**HR**	**95% CI**	**HR**	**95% CI**
**All women**	6246	131	4471	141	1.54	1.21 to 1.97	1.26	0.99 to 1.62
Subtypes defined by ER/PR/HER2								
TN	1341	46	1381	57	1.21	0.81 to 1.83	1.08	0.71 to 1.64
Luminal A	3853	56	2235	47	1.52	1.01 to 2.28	1.23	0.81 to 1.86
Luminal B	643	16	481	14	1.67	0.67 to 4.19	0.98	0.27 to 3.58
HER2-enriched	408	13	375	23	2.27	0.87 to 5.93	1.95	0.71 to 5.37
Subtypes defined by ER/PR/HER2/p53								
TN/p53-	825	24	664	30	1.38	0.76 to 2.51	1.32	0.70 to 2.47
TN/p53+	516	22	717	27	1.04	0.56 to 1.93	1.03	0.55 to 1.95
Luminal A/p53-	3262	39	1814	33	1.50	0.92 to 2.44	1.22	0.74 to 2.04
Luminal A/p53+	591	17	421	14	1.10	0.40 to 2.97	0.81	0.28 to 2.39
**Younger women (ages 35–49 yrs)**	3066	83	2248	84	1.45	1.06 to 1.99	1.21	0.87 to 1.66
Subtypes defined by ER/PR/HER2								
TN	861	33	785	39	1.30	0.80 to 2.12	1.11	0.67 to 1.84
Luminal A	1623	31	1069	22	1.16	0.65 to 2.05	1.01	0.56 to 1.80
Subtypes defined by ER/PR/HER2/p53								
TN/p53-	513	17	372	19	1.38	0.67 to 2.85	1.39	0.66 to 2.95
TN/p53+	349	16	413	20	1.14	0.56 to 2.35	1.07	0.51 to 2.25
Luminal A/p53-	1329	23	792	12	0.86	0.41 to 1.80	0.77	0.36 to 1.63
Luminal A/p53+	294	8	277	10	1.04	0.23 to 4.63	0.80	0.17 to 3.72
**Older women (ages 50–64 yrs)**	3181	48	2223	57	1.71	1.16 to 2.53	1.38	0.93 to 2.04
Subtypes defined by ER/PR/HER2								
TN	480	13	596	18	1.00	0.47 to 2.10	1.03	0.48 to 2.20
Luminal A	2231	25	1167	25	2.07	1.16 to 3.70	1.51	0.83 to 2.74
Subtypes defined by ER/PR/HER2/p53								
TN/p53-	313	7	292	11	1.56	0.52 to 4.67	1.34	0.41 to 4.37
TN/p53+	167	6	304	7	0.79	0.23 to 2.71	0.87	0.24 to 3.14
Luminal A/p53-	1934	16	1022	21	2.53	1.27 to 5.04	1.89	0.93 to 3.86
Luminal A/p53+	297	9	144	4	1.16	0.31 to 4.35	0.90	0.15 to 5.48

HRs are from multivariable Cox proportional hazards regression models using age (in days) at diagnosis and at death or end of follow-up as the time scale and stratified by single years of age at diagnosis. ^a^Adjusted for study site. ^b^Additionally adjusted for tumor stage. Abbreviations: HR, hazard ratio; CI, confidence interval. ER, estrogen receptor; PR, progesterone receptor; HER, human epidermal growth factor receptor; TN, triple negative. Note: TN = ER-/PR-/HER2-, Luminal A = ER+ or PR+ plus HER2-, Luminal B = ER+ or PR+ plus HER2+, HER2-enriched = ER-/PR-/HER2+.

Since black women are more likely than white women to be diagnosed with advanced stages of breast cancer, which is associated with a higher risk of mortality [50], we additionally controlled for tumor stage in our analysis. Then, the observed black-white differences in breast cancer-specific mortality were attenuated. The HR for black-white difference in older women diagnosed with luminal A/p53- breast cancer decreased from 2.53 (95% CI, 1.27 to 5.04) to 1.89 (95% CI, 0.93 to 3.86).

### Black-white difference in all-cause mortality

Similar to the results for breast cancer-specific mortality, the black-white difference in all-cause mortality risk after controlling for age at diagnosis and study site was observed among older women with luminal A tumors (HR, 2.21; 95% CI, 1.40 to 3.47, Table 3), but not among younger women diagnosed with luminal A tumor or among women diagnosed with TN tumor regardless of age group. When further stratified by p53 protein expression status, the black-white difference in all-cause mortality was observed only among older women diagnosed with luminal A/p53- breast cancer (HR, 2.49; 95% CI, 1.47 to 4.22).

**Table 3:** Adjusted HRs of all-cause mortality associated with race (black women vs. white women)

	**White**		**Black**		**Model 1**^**a**^		**Model 2**^**a,b**^	
	**Person-years**	**Death (No.)**	**Person-years**	**Death (No.)**	**HR**	**95% CI**	**HR**	**95% CI**
**All women**	6246	155	4471	180	1.65	1.32 to 2.06	1.42	1.13 to 1.78
Subtypes defined by ER/PR/HER2								
TN	1341	50	1381	66	1.25	0.85 to 1.84	1.12	0.76 to 1.66
Luminal A	3853	75	2235	70	1.75	1.25 to 2.46	1.54	1.09 to 2.18
Luminal B	643	16	481	16	1.74	0.70 to 4.31	1.07	0.32 to 3.53
HER2-enriched	408	14	375	28	2.69	1.06 to 6.85	2.41	0.90 to 6.40
Subtypes defined by ER/PR/HER2/p53								
TN/p53-	825	26	664	34	1.45	0.82 to 2.54	1.38	0.77 to 2.49
TN/p53+	516	24	717	32	1.04	0.58 to 1.88	1.04	0.57 to 1.89
Luminal A/p53-	3262	55	1814	52	1.73	1.16 to 2.58	1.62	1.08 to 2.43
Luminal A/p53+	591	20	421	18	1.45	0.60 to 3.54	0.99	0.37 to 2.70
**Younger women (ages 35–49 yrs)**	3066	90	2248	95	1.49	1.10 to 2.01	1.27	0.94 to 1.73
Subtypes defined by ER/PR/HER2								
TN	861	34	785	43	1.36	0.84 to 2.18	1.16	0.71 to 1.89
Luminal A	1623	36	1069	28	1.33	0.79 to 2.23	1.20	0.71 to 2.03
Subtypes defined by ER/PR/HER2/p53								
TN/p53-	513	18	372	21	1.44	0.72 to 2.87	1.43	0.70 to 2.90
TN/p53+	349	16	413	22	1.21	0.60 to 2.47	1.13	0.54 to 2.36
Luminal A/p53-	1329	28	792	17	1.06	0.56 to 2.01	1.05	0.54 to 2.02
Luminal A/p53+	294	8	277	11	1.32	0.32 to 5.42	0.92	0.21 to 4.06
**Older women (ages 50–64 yrs)**	3181	65	2223	85	1.89	1.36 to 2.62	1.62	1.17 to 2.26
Subtypes defined by ER/PR/HER2								
TN	480	16	596	23	1.04	0.54 to 2.02	1.07	0.55 to 2.11
Luminal A	2231	39	1167	42	2.21	1.40 to 3.47	1.88	1.18 to 2.99
Subtypes defined by ER/PR/HER2/p53								
TN/p53-	313	8	292	13	1.66	0.61 to 4.54	1.48	0.50 to 4.41
TN/p53+	167	8	304	10	0.77	0.27 to 2.20	0.79	0.27 to 2.30
Luminal A/p53-	1934	27	1022	35	2.49	1.47 to 4.22	2.22	1.30 to 3.79
Luminal A/p53+	297	12	144	7	1.54	0.48 to 4.91	1.44	0.25 to 8.11

HRs are from multivariable Cox proportional hazards regression models using age (in days) at diagnosis and at death or end of follow-up as the time scale and stratified by single years of age at diagnosis. ^a^Adjusted for study site. ^b^Additionally adjusted for tumor stage. Abbreviations: HR, hazard ratio; CI, confidence interval. ER, estrogen receptor; PR, progesterone receptor; HER, human epidermal growth factor receptor; TN, triple negative. Note: TN = ER-/PR-/HER2-, Luminal A = ER+ or PR+ plus HER2-, Luminal B = ER+ or PR+ plus HER2+, HER2-enriched = ER-/PR-/HER2+.

The observed black-white differences in all-cause mortality were also decreased after additionally controlling for tumor stage, but the magnitude of the decrease appeared smaller than that observed for breast cancer-specific mortality. The HR for black-white difference in all-cause mortality in older women diagnosed with luminal A/p53- breast cancer decreased from 2.49 (95% CI, 1.47 to 4.22) to 2.22 (95% CI, 1.30 to 3.79).

### Test for homogeneity across subtypes

Although black-white differences in mortality after breast cancer diagnosis were observed only among older women diagnosed with luminal A and luminal A/p53- subtype, no tests for homogeneity of race-specific HRs across subtypes achieved statistical significance (results not shown).

## Discussion

In the current analysis of 1,204 women 35 to 64 years of age, with a median follow-up of 10 years, we did not observe any statistically significant black-white differences in cancer-specific or all-cause mortality among women diagnosed with TN subtype. We did, however, find that black women had statistically significant greater all-cause mortality risk than white women among those ages 50–64 years who were diagnosed with luminal A tumors, and more specifically among those diagnosed with luminal A/p53- breast cancer. However, no tests for homogeneity of race-specific HRs comparing luminal A to TN subtype and luminal A/p53- to luminal A/p53+ subtype achieved statistical significance.

The results from four previous epidemiologic studies that compared mortality risk or survival in black and white women diagnosed with luminal A or TN or basal-like subtype are inconsistent [4-7]. One study with 11 to 13 years of follow-up of 476 (116 black, 360 white) Atlanta women diagnosed between 1990 and 1992 with invasive breast cancer at ages 20–54 years found that risk of all-cause mortality was greater among black women than among white women for both luminal A cancer (unadjusted HR, 1.6; 95% CI, 1.1 to 2.4) and TN breast cancer (unadjusted HR, 2.1; 95% CI, 1.3 to 3.3). The racial difference disappeared for luminal A breast cancer after adjustment for age, stage, and grade (adjusted HR, 1.1; 95% CI, 0.7 to 1.6), whereas it persisted for TN breast cancer even after additional adjustment for poverty level, treatment, and comorbidities (adjusted HR, 2.0; 95% CI, 1.0 to 3.7) [4]. A second, smaller study followed 124 (88 black, 36 white) women ages 26–82 years with invasive TN breast cancer treated at the University of Tennessee Cancer Institute, Memphis, between 2003 and 2008 for a median of 23 months [6]. Older black breast cancer patients (≥55 years at diagnosis) with TN breast cancer had poorer breast cancer-specific survival than older white women. A third study compared 331 lower income AA women with 203 lower income non-AA women consisting of 115 Hispanic and 88 non-Hispanic white women, who were treated for breast cancer at a large urban public hospital providing care to the medically uninsured in metropolitan Chicago between 2000 and 2005 [7]. This study found that AA women had a higher crude all-cause mortality risk than non-AA women (HR, 1.45; 95% CI, 1.03 to 2.05) irrespective of the subtypes defined by ER, PR, and HER2 status. Results from the Carolina Breast Cancer Study, which followed 1,149 (518 black, 631 white) women with invasive breast cancer from diagnosis between 1993 and 2001 through 2006, are consistent with our results. This study found that the black-white difference in breast cancer-specific mortality was observed for women diagnosed with luminal A breast cancer, but not for those diagnosed with basal-like (ER-/PR-/HER2- plus HER1+ and/or CK 5/6+) breast cancer (age-, date of diagnosis-, and stage at diagnosis-adjusted HR, 1.9; 95% CI, 1.3 to 2.9 and HR, 1.3; HR, 0.8 to 2.3 for luminal A and basal-like breast cancer, respectively) [5].

An analysis comparing the outcomes of 405 black women with 4,412 nonblack women who had stage I-III breast cancer and who participated in a National Cancer Institute-sponsored randomized phase III trial also provides supporting evidence for our results [51]. Breast cancer-specific and overall survival was lower in black women with luminal A disease than in nonblack women, but no racial differences were observed for women with other subtypes of breast cancer.

Based on our knowledge, this is the first study to examine if the overexpression status of p53 protein impacts the black-white disparities in mortality of TN or luminal A breast cancer. Our data showed that p53 protein expression status could impact black-white mortality differences, and this was most evident for older women diagnosed with luminal A breast cancer. A possible explanation for no black-white difference in mortality risk for older women with luminal A/p53+ tumor is that luminal A/p53+ tumor is currently less likely to be treatable for either black women or white women since mutations in *p*53 are associated with resistance to chemotherapy, radiotherapy, and poor prognosis [31,32]. The reasons for a statistically significantly higher risk in all-cause mortality rather than in breast cancer-specific mortality in older black women diagnosed with luminal A/p53- tumor than their white counterparts, could be related to several adverse factors for overall survival, such as more comorbidities [7,52] and less access to adequate health care because of lower socioeconomic status [53]. The adjustments for all these factors could attenuate the observed black-white difference in all-cause mortality risk. Unfortunately, we have data only for potential comorbidities diagnosed prior to breast cancer and for education which can serve as as a rough proxy for social economic status. In our study, the HR for black-white difference in all-cause mortality in older women diagnosed with luminal A/p53- breast cancer decreased from 2.22 (95% CI, 1.30 to 3.79) to 1.64 (0.90 to 3.01) and the HR for black-white difference in breast cancer-specific mortality in older women diagnosed with luminal A/p53- breast cancer decreased from 1.89 (95% CI, 0.93 to 3.86) to 1.50 (95% CI, 0.66 to 3.43), after additionally adjusting for the number of comorbidities (zero, one, two or more including hypertension, myocardial infarction, stroke, diabetes, and cancers other than nonmelanoma skin cancers) and education (≤high school, technical school/some college, college graduate; results not shown).

This study had several limitations. First, we were unable to request tissue for all eligible women diagnosed with invasive breast cancer in the two study sites because of funding constraints. However, we obtained paraffin-embedded tissue for 80% of the samples requested. Second, we did not have breast cancer treatment information available and therefore did not adjust for treatments in our analyses. Although we have presumed that controlling for age, stage of disease, and the status of the four tumor markers has provided some control for treatment, previous studies have reported that black women may receive less optimal treatment than white women [54-58]. Black women are more likely to delay the initiation of treatment [54], less likely to receive surgery [55] or optimal adjuvant systemic therapy [56], less likely to adhere to recommended treatment regimens [57], and more likely to terminate treatment prematurely [58] than white women. If any black-white differences in treatment existed in our participants, the HRs for a black-white difference in mortality risk could be overestimated, but it is unlikely that this bias would differ across tumor subtypes. Third, although our HRs for a black-white difference in both breast cancer-specific and all-cause mortality suggest that a large black-white difference in mortality risk may exist in women diagnosed with HER2-enriched tumors, the number of deaths was limited for this analysis. Fourth, due to funding limitations, we evaluated p53 protein expression, but not *p*53 mutations. Although previous research shows that p53 protein expression and *p*53 mutation status determined by FISH analysis are strongly correlated, our assessment of p53 protein expression by IHC may have misclassified some tumors. Fifth, although the agreement in the classification for ER and PR status between the SEER registry and centralized laboratory was substantial [59], we repeated the analyses for TN and luminal A and their subtypes defined by p53 status using ER/PR status from SEER instead of those from the centralized laboratory for the 918 women who had both ER and PR expression status in SEER; we obtained similar results (data not shown). Finally, our study provides evidence suggesting that black-white differences in mortality vary by tumor subtypes among older women. However, the number of deaths among older black women with TN subtype was small resulting in limited statistical power to detect statistically significant difference in race-specific HRs between luminal A and TN breast cancer. The number of deaths among older black women with luminal A/p53+ subtype was also small resulting in limited statistical power to detect significant difference in race-specific HRs between luminal A/p53- and luminal A/p53+ subtype. Therefore, confirmation of our results will require larger studies to demonstrate statistically meaningful differences.

## Conclusions

Our findings suggest that the black-white difference in mortality risk is mainly among women 50 years or older diagnosed with luminal A/p53- breast cancer, a subtype for which treatments exist. These results further suggest that factors other than subtype may be relatively more important in explaining the increased mortality risk seen in older black women.

## Abbreviations

AA: African American; BMI: Body mass index; ER: Estrogen receptor; PR: Progesterone receptor; HER: Human epidermal growth factor receptor; TN: Triple negative; CARE: Contraceptive and reproductive experiences; LA: Los Angeles; IHC: Immunohistochemistry; FISH: Fluorescent in situ hybridization; HR: Hazard ratio; CI: Confidence interval.

## Competing interests

The authors declare that they have no competing interests.

## Authors’ contributions

RS, DMD, BLS, and LB participated in the study design and supervised the collection and assembly of data. KEM, PAM, RTB, JAM, SGF, and JS supervised or participated in the collection and assembly of data. HM conducted data analyses and drafted the manuscript with LB’s input. All authors participated in the revision of the manuscript and have read and approved the final version.

## Pre-publication history

The pre-publication history for this paper can be accessed here:

http://www.biomedcentral.com/1471-2407/13/225/prepub
